# Mesenchymal gene expression subtyping analysis for early-stage human papillomavirus-negative head and neck squamous cell carcinoma reveals prognostic and predictive applications

**DOI:** 10.3389/fonc.2022.954037

**Published:** 2022-09-06

**Authors:** Gregory M. Mayhew, Joshua M. Uronis, David Neil Hayes, Jose P. Zevallos

**Affiliations:** ^1^ Department of Bioinformatics and Biostatstics, GeneCentric Therapeutics Inc., Durham, NC, United States; ^2^ Department of Genomics Sequencing Operations, GeneCentric Therapeutics Inc., Durham, NC, United States; ^3^ Department of Medical Oncology, University of Tennessee Health Sciences West Cancer Center, Memphis, TN, United States; ^4^ Department of Otolaryngology-Head and Neck Surgery , Washington University School of Medicine in St. Louis, MO, United States

**Keywords:** head and neck, oral cavity, HPV-negative, mesenchymal, gene expression

## Abstract

Patients with oral cavity squamous cell carcinoma (OCSCC) are predominantly human papillomavirus (HPV)(−), and treatment typically involves surgical resection ± neck dissection, followed by radiation ± chemotherapy. We previously described four mRNA expression patterns (classical, atypical, basal, and mesenchymal), each with unique genomic features and prognosis. Here, we examine the clinical utility of gene expression subtyping in head and neck squamous cell carcinoma (HNSCC) and introduce potentially predictive applications in HPV(−) OCSCC. A retrospective genomic database analysis was performed including 562 HNSCC patients from MD Anderson (MDA-GSE41116) and The Cancer Genome Atlas (TCGA). Samples were assigned molecular subtypes (classical, atypical, basal, and mesenchymal) using an 88-gene classifier. HPV status was determined by gene expression. The clinical endpoint was overall survival censured at 36 months. The Kaplan–Meier plots and log-rank tests were used to investigate associations between clinical variables and survival. Of the 418 TCGA training patients who met analysis criteria, nearly 20% presented as stage I/II. Among node(−) OCSCC patients, the mesenchymal subtype is associated with worse survival (hazard ratio (HR) = 2.4, p = 0.021), offering a potentially actionable biomarker in otherwise early-stage, low-risk disease. This was confirmed in the MDA validation cohort. Node(−) non-mesenchymal OCSCC patients had far better survival compared to node(−) mesenchymal, and all node(+) patients had similarly poor survival. These findings suggest that the mesenchymal subtype is associated with poor survival in surgically resected, early-stage, node(−) OCSCC otherwise expected to have favorable outcomes. These findings highlight the potential value of gene expression subtyping as a pathology adjunct for prognostication and treatment decision-making in OCSCC patients.

## Introduction

Head and neck squamous cell carcinoma (HNSCC), including cancers of the oral cavity, oropharynx, nasopharynx, hypopharynx, and larynx, is one of the most common cancers worldwide (1). In the United States, it is estimated that there were approximately 66,000 new cases and 14,00 deaths in 2021 (1). The majority of HNSCC cases are associated with heavy tobacco and alcohol use, although over the last 30 years, there has been an increase in the incidence of human papillomavirus (HPV)-related cancers, primarily in the oropharynx. While treatment of HNSCC depends on multiple tumor and patient-related factors, the three main modalities used in HNSCC management are surgical resection, radiation therapy, and chemotherapy. Patients with early-stage tumors are generally treated with a single modality therapy, while those with advanced-stage tumors often require multiple modalities. Oncologic outcomes in HNSCC are driven largely by stage at presentation: the 5-year overall survival for stage I–II and III–IV HNSCC cases is approximately 70%–90% and 40%–60%, respectively.

While the majority of early-stage HNSCC cases are curable with surgery or radiation, it is notable that 10%–30% of HPV-negative HNSCC cases without pathologically aggressive features still experience a relapse event (2). Oral cavity squamous cell carcinoma (OCSCC or OC) is the most common head and neck cancer, comprising 1/3 of all cases, with the vast majority of cases presenting as HPV-negative and associated with tobacco use. Dependent on clinical staging, OC treatment involves surgical excision of the primary tumor with or without neck dissection, followed by radiation with or without chemotherapy.

There have been significant advances in our understanding of HNSCC molecular biology and genomic tumor heterogeneity. Based on earlier work in lung cancer (3), our group and others described four mRNA expression patterns (classical, atypical, basal, and mesenchymal) demonstrating unique genomic features and prognostic significance (4, 5). These subtypes show varied biology and may be helpful in prognostic assessments complementing risk stratification based on HPV status, stage, anatomic site, and other characteristics (4, 5). The basal subtype is characterized by over-expression of genes functioning in cell adhesion including *COL17A1*, and growth factor and receptor *TGFA* and *EGFR* (5). The mesenchymal subtype displays over-expression of genes involved in immune response (6, 7) and is characterized by the expression of genes associated with epithelial-to-mesenchymal transition (EMT) including *VIM*, *DES*, *TWIST1*, and *HGF (* 5). It has been suggested previously that EMT pathways are important in the initiation of nodal metastasis (8). The classical subtype is characterized by over-expression of genes related to oxidative stress response and xenobiotic metabolism and is most strongly associated with tobacco exposure (9–12). The atypical subtype, which includes both HPV and non-HPV tumors, is characterized by elevated expression of *CDKN2A*, *LIG1*, and *RPA2* and is associated with low EGFR expression (5). These four gene-expression-based head and neck cancer subtypes have been validated in other studies, including The Cancer Genome Atlas (TCGA) head and neck cancer cohort (4–6).

In this study, we examine the potential clinical utility of gene expression subtyping in HNSCC, with an emphasis on evaluating this biomarker among early-stage HPV-negative cancers. Our findings provide further evidence to support the clinical utility of gene expression subtyping in HNSCC within the context of clinical site, stage, and treatment as well as to introduce the potential for predictive applications of gene expression subtyping analysis in HPV-negative HNSCC.

## Methods

### Patients and datasets

The study was conducted in accordance with the Declaration of Helsinki and the International Conference on Harmonization Good Clinical Practice guidelines and was approved by the Institutional Review Boards of Washington University in St. Louis (IRB#201706088) and the University of Tennessee Health Science Center (IRB# 17-05549-XP).

Two datasets were identified in the public record: 1) TCGA HNSCC (n = 520) (6) and 2) large institutional cohort (n = 42) (13). For statistical analyses, cases were considered if they had clinical parameters of N stage and overall survival. Model fits and analyses used all available patients having complete relevant data. Patient statistics and demographics are shown in **Table 1**. TCGA data were sourced from the Broad Institute Genomic Data Commons (GDC) (14), and the institutional cohort was obtained from the gene expression omnibus (GEO) (GSE41116) (15).

**Table 1:** Descriptive Statistics of Clinical and Demographic Variables by Molecular Subtype (n=418).

Oral Cavity							Non-Oral Cavity						All Samples
	N0		P-Value^1^	N+		P-Value^2^	N0		P-Value^3^	N+		P-Value^4^	Total
	Mesenchymal(n=26)	Non-Mesenchymal(n=93)		Mesenchymal(n=53)	Non-Mesenchymal(n=97)		Mesenchymal(n=10)	Non-Mesenchymal(n=46)		Mesenchymal(n=20)	Non-Mesenchymal(n=73)		
Location													
Oral Cavity	26(100%)	93(100%)		53(100%)	97(100%)								269
Larynx							6(60%)	34(74%)		13(65%)	42(58%)		95
Oropharynx							3(30%)	12(26%)		4(20%)	28(38%)		47
Hypopharynx							1(10%)	0(0%)		3(15%)	3(4%)		7
Age (years)													
Median	66	62		64	58	0.014	61.5	60.5		60.5	59		60
Gender													
F	9(35%)	36(39%)		15(28%)	22(23%)		1(10%)	9(20%)		2(10%)	13(18%)		107
M	17(65%)	57(61%)		38(72%)	75(77%)		9(90%)	37(80%)		18(90%)	60(82%)		311
Smoker (ever)													
Yes	16(67%)	61(66%)		41(80%)	73(77%)		9(90%)	41(91%)		15(79%)	65(89%)		321
No	8(33%)	31(34%)		10(20%)	22(23%)		1(10%)	4(9%)		4(21%)	8(11%)		88
HPV status													
Positive	1(4%)	7(8%)		5(9%)	14(14%)		1(10%)	9(20%)		2(10%)	24(33%)		63
Negative	25(96%)	86(92%)		48(91%)	83(86%)		9(90%)	37(80%)		18(90%)	49(67%)		355
Radiation													
Yes	10(40%)	40(45%)		30(62%)	65(76%)		5(56%)	19(49%)		11(73%)	57(88%)		237
No	15(60%)	49(55%)		18(38%)	20(24%)		4(44%)	20(51%)		4(27%)	8(12%)		138
T stage													
T1	3(12%)	16(17%)	0.034	3(6%)	5(5%)		3(30%)	4(9%)		1(5%)	7(10%)		42
T2	15(58%)	24(26%)		14(26%)	26(27%)		1(10%)	12(27%)		4(20%)	16(22%)		112
T3	3(12%)	18(19%)		14(26%)	23(24%)		2(20%)	7(16%)		3(15%)	25(35%)		95
T4	5(19%)	35(38%)		22(42%)	43(44%)		4(40%)	22(49%)		12(60%)	24(33%)		167
N stage													
N0	26(100%)	93(100%)		0(0%)	0(0%)		10(100%)	46(100%)		0(0%)	0(0%)		175
N1	0(0%)	0(0%)		16(30%)	31(32%)		0(0%)	0(0%)		5(25%)	15(21%)		67
N2	0(0%)	0(0%)		36(68%)	64(66%)		0(0%)	0(0%)		14(70%)	54(74%)		168
N3	0(0%)	0(0%)		1(2%)	2(2%)		0(0%)	0(0%)		1(5%)	4(5%)		8
M stage													
M0	9(100%)	40(100%)		22(100%)	42(100%)		8(100%)	24(100%)		9(100%)	24(96%)		178
M1	0(0%)	0(0%)		0(0%)	0(0%)		0(0%)	0(0%)		0(0%)	1(4%)		1
Overall stage													
I	3(12%)	15(16%)	0.037	0(0%)	0(0%)		3(30%)	3(7%)		0(0%)	0(0%)		24
II	15(58%)	24(26%)		0(0%)	0(0%)		1(10%)	13(29%)		0(0%)	1(1%)		54
III	3(12%)	18(20%)		11(21%)	23(24%)		2(20%)	7(16%)		2(10%)	11(15%)		77
IV	5(19%)	35(38%)		42(79%)	73(76%)		4(40%)	22(49%)		18(90%)	59(83%)		258
Subtype													
Basal		57(61%)			52(54%)			9(20%)			6(8%)		124
Mesenchymal	26(100%)			53(100%)			10(100%)			20(100%)			109
Atypical		20(22%)			23(24%)			26(57%)			40(55%)		109
Classical		16(17%)			22(23%)			11(24%)			27(37%)		76

Statistical comparisons: 1oral cavity, N0, mesenchymal vs. oral cavity, N0, non-mesenchymal. 2Oral cavity, N+, mesenchymal vs. oral cavity, N+, non-mesenchymal. 3Non-oral cavity, N0, mesenchymal vs. non-oral cavity, N0, non-mesenchymal. 4Non-oral cavity, N+, mesenchymal vs. non-oral cavity, N+, non-mesenchymal. Some variables do not sum to total due to missing data.

### Gene expression analysis

For TCGA, the upper quantile normalized RNA-seq expression values by expected maximization (RSEM) (16) were downloaded from GDC (gdac.broadinstitute.org/, accessed 12/4/2015) and log2-transformed. All samples were assigned a molecular subtype using the nearest centroid classification method as previously reported by Dabney (17), describing each sample as belonging to one of four molecular subtypes (basal, mesenchymal, atypical, or classical). Briefly, the HNSCC centroid predictor is a set of vectors, each comprised of typical gene expression values for one of the subtypes, and uses a set of 838 feature genes selected to distinguish the four molecular subtypes (5). By calculating the distance (1 − Pearson correlation coefficient) between each sample and each centroid, the algorithm determines the subtype to which the sample is most similar based on the predictor gene set. Each sample is then uniquely assigned to the subtype to which the distance was the shortest. For the purpose of developing a more parsimonious and potentially clinically relevant predictor, a reduced, 88-gene centroid predictor was developed (**Supplementary Table 1**). Fivefold cross-validation using all 520 samples and the ClaNC software package was used to identify the number of genes required for strong separation of the subtypes and sufficient agreement with the previously developed gold standard. Candidates for the reduced set were all genes in the gold standard classifier, and an additional set of genes (348) was chosen for high observed mean and variance in the entire data set. Here the standard ClaNC approach was modified by requiring an equal proportion of high and low genes per subtype in the final model rather than selecting genes based on extreme absolute values of the ClaNC t-statistic. Calculation of the coefficients in the final nearest centroid classifier excluded samples with low gold standard classifier call strength (20% per subtype were excluded), where call strength was the commonly used silhouette score, and the coefficients themselves were within-subtype gene medians after each gene had been centered by its overall median. Heat maps displaying expression profile patterns by subtype calls were generated using the Complexheatmap package in R.

### Clinical data and statistical analysis

Paired clinic data were obtained from GDC (gdac..broad.instituteorg/accessed 12/4/2015) (14). To account for limitations in median follow-up, and for comparison to prior work, all survival times longer than 36 months were truncated and censored at 36 months. In general, clinical parameters were represented as presented in downloaded clinical datasets. HPV positivity was assessed by RNA-seq evaluation of HPV-aligned sequences in HPV types 16, 18, 33, and 35 at levels >1,000 counts. HPV reference sequence data were based on the PaVe website: https://pave.niaid.nih.gov/. Read counts >1,000 for HPV RNA-seq (TCGA) or HPV E6 gene expression (18) were used as the criterion for ongoing HPV replication and an HPV-positive tumor designation. Other parameters of interest included gender, age, smoking, T stage, N stage, and overall stage. Associations between two categorical variables were evaluated using Fisher’s exact test and the chi-square test. Associations between categorical and continuous variables were evaluated using the Kruskal–Wallis test. The Kaplan–Meier plots and the log-rank test were used to assess univariate associations between survival and study parameters. Cox models were used to check associations with adjustment for potential confounders. The R survival package was used for all statistical analyses.

## Results

### Clinical and molecular groups

We first considered the clinical characteristics of 418 patients from TCGA dataset meeting our eligibility criteria to understand the generalizability of our results to the greater population of head and neck cancers. The median age of TCGA HNSCC cohort was 60 years, only slightly younger than that reported in the American population for this disease, which is 63 years (1). Twenty-five percent of patients were female compared to 27% of patients in the American HNSCC population. Seventy-eight percent of patients in the dataset admitted some degree of smoking, which is consistent with prior reports (19). Eighty-one percent of patients presented with at least stage III disease, consistent with most datasets studied by molecular profiling. Consistent with the head and neck cancer disease course, only one patient was known to have metastatic disease at presentation, although this data field was missing for more than half of the patients. Larger, more advanced tumors tend to yield more sufficient tissue for molecular profiling. Nonetheless, nearly 20% of patients presented as stage I and II, offering a unique opportunity to assess risk profiles in early-stage patients. Somewhat unexpectedly, only 63% of patients had a record of radiation in the dataset, which seems low considering that most of the 81% of advanced stage patients might be expected to receive radiation as part of multidisciplinary treatment. Considering subgroups, we noted that 71% of node-positive oral cavity patients reported radiation versus 85% of the node-positive non-oral cavity. Forty-three percent of node-negative oral cavity patients were radiated, compared to 50% of the node-negative non-oral cavity. Whether the low percentage represents underutilization of the standard of care based on patient-specific factors or under-reporting of radiation in the database is not known. That said, the trends were as expected in which the highest rates of radiation were found in node-positive non-OC patients, for whom nearly all patients would have a recommendation for radiation based on National Comprehensive Cancer Network (NCCN) guidelines, either as part of concurrent chemoradiation or surgery followed by radiation. The lowest rates were among node-negative oral cavity patients, many of whom could be treated with single modality surgery, according to NCCN guidelines.

**Figure 1:**
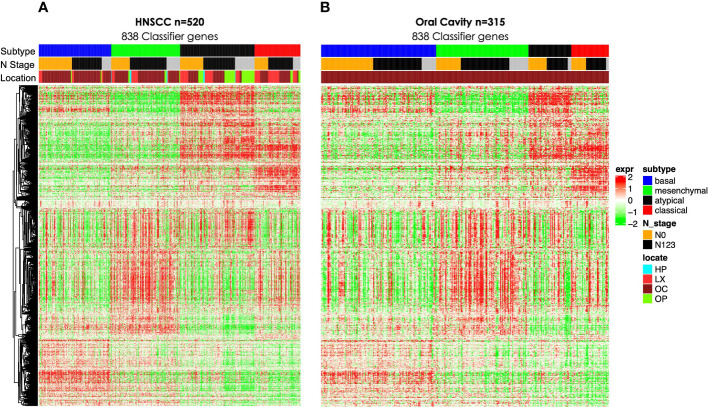
Gene expression heat maps including 838 gene classifier genes as described previously (5) for **(A)** all TCGA HNSCC (n = 520) and **(B)** OC (n = 315). TCGA, The Cancer Genome Atlas; HNSCC, head and neck squamous cell carcinoma; OC, oral cavity.

**Figure 2:**
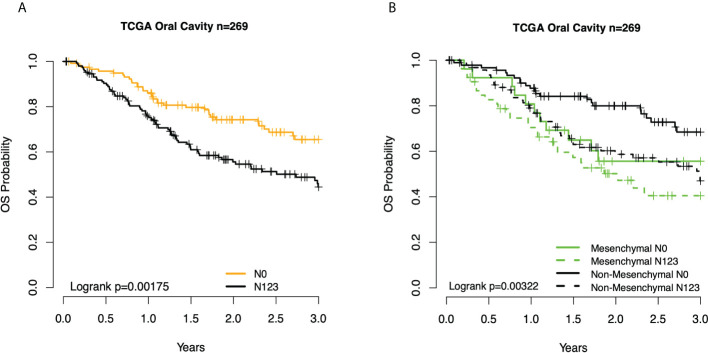
Kaplan–Meier overall survival (OS) curves for TCGA OC patients. **(A)** By node status. **(B)** Subtype and node status group. TCGA, The Cancer Genome Atlas; OC, oral cavity.

**Figure 3:**
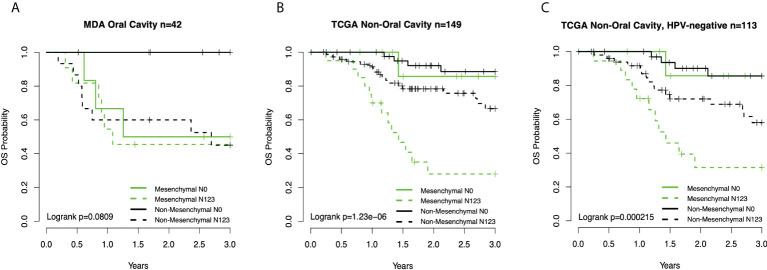
**(A)** Kaplan–Meier overall survival (OS) curves for oral cavity cancer patients (13) by subtype-node status group. **(B)** Curves for TCGA non-OC patients by subtype-node status. **(C)** Curves TCGA non-OC HPV-negative patients by subtype-node status group. TCGA, The Cancer Genome Atlas; OC, oral cavity; HPV, human papillomavirus.

We then interrogated the distribution of molecular subtypes as a function of the anatomic site, where subtypes were determined by applying the centroid subtype predictor to all samples. We document that in the 418 TCGA samples meeting eligibility criteria, the distribution of molecular subtypes was nearly identical to that of the original TCGA HNSCC report of 279 cases published in 2015: 30% basal, 26% mesenchymal, 18% classical, and 26% atypical versus 31% basal, 27% mesenchymal, 16% classical, and 26% atypical (6) (**Figure 1A** and **Table 1**). Consistent with other reports, there is a strong association of subtype with the anatomic site. We observed enrichment in oral cavity tumors among the mesenchymal and basal subtypes, atypical samples primarily in the oropharynx and to a lesser extent the larynx, and classical subtype in the larynx. In an unexpected finding, we found that although lymph node involvement is observed in all molecular and anatomic sites as expected, we observed a statistically significant association with lymph node positivity in the mesenchymal tumors (p = 0.03), where the proportion of positive nodes in mesenchymal was 67% compared to 55% in non-mesenchymal subtypes. Overall, node-negative OC patients demonstrated significantly better survival than node positive OC patients (**Figures 2A**), however we also found a significant association between Mesenchymal- and non-mesenchymal subtype and overall survival in OC, which our group and others had previously reported in smaller cohorts as a statistically significant association for overall survival and mesenchymal subtype (4–6, 13). The finding was again observed in this cohort (**Table 2**) (**Figures 2B**, **3**). Since nodal status is a component of the overall cancer stage, itself defining patient prognosis, we considered that lymph node involvement might either be confounding for the worse prognosis for mesenchymal tumors or, more interestingly, be in the causal pathway of poor prognosis.

**Table 2:** Univariate and multivariate survival analysis within Oral Cavity N0 and N+ subgroups.

Oral Cavity NO								
			Univariate			Multivariate		
	Reference	n	HR	Cl	P-Value	HR	Cl	P-Value
Gender	Female	119	0.98	(0.49,1.99)	0.96			
Smoker	No	116	0.78	(0.38,1.59)	0.49			
HPV Status	Positive	119	1.24	(0.3,5.17)	0.77			
Radiation	Yes	114	1.94	(0.92,4.1)	0.082			
T stage	1,2	119	2.3	(1.12,4.72)	0.023	2.77	(1.32,5.82)	0.0072
Overall Stage	I, II	118	2.48	(1.18,5.21)	0.017			
Subtype	Non-Mesenchymal	119	1.83	(0.89,3.76)	0.099	2.4	(1.14,5.06)	0.021
Age	19-61	119	0.95	(0.48,1.88)	0.88			
**Oral Cavity N+**								
	** **		**Univariate **			**Multivariate**		
	**Reference**	**n**	**HR**	**Cl**	**P-Value**	**HR**	**Cl**	**P-Value**
Gender	Female	150	1.06	(0.61,1.84)	0.83			
Smoker	No	146	2.09	(1.03,4.22)	0.041			
HPV	Positive	150	1	(0.49,2.01)	0.99			
Radiation	Yes	133	2.12	(1.25,3.6)	0.0053			
T stage	1,2	150	2.33	(1.25,4.34)	0.008			
Overall Stage	I, II	149	2.44	(1.17,5.11)	0.018			
Subtype	Non-Mesenchymal	150	1.36	(0.84,2.21)	0.22	1.3	(0.79,2.14)	0.3
Age	19-61	150	1.3	(0.81,2.1)	0.28	1.24	(0.76,2.01)	0.39

HR, hazard ratio; CI, confidence interval.

### Oral cavity cohort

For the purposes of defining a cohort in which questions of clinical management and prognosis might be more specifically considered, we isolated patients with oral cavity squamous cancers (**Figure 1B**), a group generally treated by a more explicitly clinical pathway. In general, patients with OC are treated primarily with surgery in all cases where a tumor is expected to be resected with negative margins. Early-stage patients, such as stages I and II, can be managed with surgery only or surgery plus adjuvant radiation. Patients with more advanced tumors generally receive surgery followed by radiation or concurrent chemoradiation. Patients with OC were primarily basal, n = 109 (41%), and mesenchymal, n = 79 (29%), with minority contributions of atypical, n = 43 (16%), and classical, n = 38 (14%). Overall, 67% (53 of 79) of the mesenchymal patients were node-positive versus 48% (52 of 109) basal, 53% (23 of 43) atypical, and 58% (22 of 38) classical. A finding of a higher probability of node involvement would be consistent with a higher clinical stage and the overall worse prognosis observed for mesenchymal patients in multiple datasets. Interestingly, although the mesenchymal patients were overall of higher nodal status, the association with the T stage was less clear. Among node-positive OC patients, mesenchymal and non-mesenchymal patients had nearly identical nodal stage distribution of N1, N2, and N3. By contrast, among node-negative patients, 69% (18 of 26) of mesenchymal patients were T1 or T2, whereas only 43% (40 of 93) of non-mesenchymal patients were T1 or T2. Only 19% (5 of 26) of node-negative mesenchymal patients were T4 compared to 38% (35 of 93) of non-mesenchymal patients. Of T1–T2 mesenchymal tumors, 49% (17 of 35) were node-positive, compared to 44% (31 of 71) of T1–T2 non-mesenchymal patients. By contrast, of mesenchymal T3–T4 tumors, 82% (36 of 44) were node-positive compared to 55% (66 of 119) of non-mesenchymal T3–T4 tumors. Additionally, in mesenchymal patients, T3–T4 tumors were associated with node positivity (p = 0.003), whereas the T stage was not associated with node status in non-mesenchymal patients (p = 0.13). Summarizing the associations for OC patients, we conclude that mesenchymal patients are both more likely to develop nodal metastasis, and they are more likely to do this at an earlier T stage. At the higher T stage, mesenchymal patients were much more likely to be node-positive.

Given the association between three clinical prognostic factors (anatomic site, and T and N stage) with a validated molecular marker (mesenchymal subtype), we considered both stratified and multivariate models of prognosis to investigate potential subgroups as well as the independent contribution of each factor (**Table 2**). We demonstrate, as expected, that clinical outcomes differ as a function of the anatomic site, T stage, and N stage. We then considered substrata as a function of nodal status, observing a striking finding in which those patients who were node-negative mesenchymal subtype demonstrated identical risk of death to patients who were node-positive mesenchymal or node-positive non-mesenchymal (hazard ratio (HR) = 2.4, p = 0.021). In other words, the mesenchymal molecular subtype conveyed all the risk of positive nodes whether nodes were clinically present or not. Among node-positive patients, the added risk of the mesenchymal subtype was no longer observed (HR = 1.3, p = 0.3). Given the unexpected nature of this finding, we sought independent datasets of oral cavity cancer for the purposes of validation, noting perhaps the largest being a set of well-characterized tumors from MD Anderson. Quite strikingly, the results were nearly identical, with patients of the non-mesenchymal OC group showing overall excellent survival and node-negative mesenchymal patients, node-positive mesenchymal patients, and node-positive non-mesenchymal patients all with similarly poor survival (**Figure 3**).

We then considered the remaining mesenchymal patients from non-OC sites, of which there were only 30 out of 418 TCGA patients, divided roughly equally between the larynx and oropharynx. The non-OC mesenchymal patients were 1/3 node-negative and 2/3 node-positive. Unlike OC patients, the node-negative patients did extremely well overall. Although the sample number was only 10 patients, there was no suggestion that non-OC mesenchymal patients did worse than non-mesenchymal patients in the node-negative state. By contrast, node-positive mesenchymal patients did poorly overall compared to node-positive non-OC patients. We then excluded HPV(+) patients, where the results were somewhat attenuated, but mesenchymal patients still fared worse than non-mesenchymal patients.

## Discussion

This study confirms several important previous findings regarding gene expression subtypes in OC and HNSCC more broadly. First, we demonstrate that the mesenchymal and basal subtypes comprise the majority of OC cases. Second, we confirm previous reports suggesting that the mesenchymal subtype is associated with worse outcomes across all HNSCC sites. Importantly, we also identify novel and more nuanced associations between gene expression subtypes, stage, site, and treatment with important implications for future treatment stratification. Remarkably, we demonstrate that the mesenchymal subtype is associated with poor survival even in the setting of early-stage, node-negative OC treated with surgical resection. In contrast, our data demonstrate that mesenchymal subtype cases have favorable outcomes compared to other gene expression subtypes in early stage, non-OC cases, the majority of which were treated with definitive radiation therapy. These findings highlight the potential value of gene expression subtyping as an adjunct to pathology for treatment decision-making.

Gene expression subtypes provide an objective method of molecular classification of HNSCC based on unsupervised clustering and are reflective of important differences in tumor biology. The four gene expression subtypes in HNSCC have been validated in multiple datasets, and similar classifications have been developed for lung cancer (3–5, 20). In the present study, we demonstrate differences in gene expression subtype distribution by anatomic site. As previously reported, OC is comprised primarily of mesenchymal and basal subtypes, while classical is the predominant subtype in laryngeal squamous cell carcinoma (LSCC). Our group and others (13) have previously demonstrated the prognostic value of the mesenchymal subtype in HNSCC. The mesenchymal subtype is associated with EMT and predisposes to increased tumor invasiveness and lymph node metastases (5, 21–23). Recently, a partial EMT signature has shown further evidence of the importance of a mesenchymal phenotype in OC, suggesting that the transition from epithelial to mesenchymal phenotype represents a spectrum.

This study provides a more refined examination of the prognostic value of gene expression subtype in HNSCC that is specific to early-stage HNSCC. While the mesenchymal subtype is prognostic of worse survival in early-stage OC, there is no significant difference in outcomes between mesenchymal and other subtypes in non-OC early-stage tumors. Therapeutic decision-making and treatment dilemmas in HNSCC are anatomic site specific, and our data suggest that the potential clinical application of molecular subtyping should be considered within this context. These data also highlight the potential predictive application of gene expression subtype analysis in HNSCC. OC is generally treated surgically, with adjuvant radiation and chemotherapy reserved for advanced-stage tumors or adverse pathologic features such as positive margins and extranodal extension. Nevertheless, there is a subset of OC patients who recur even with early-stage disease and in the absence of adverse pathologic features (2). We have previously shown that the mesenchymal subtype is associated with an increased risk of occult nodal metastasis in the setting of clinically node-negative disease and suggest that a gene expression classifier applied to early stage HNSCC could potentially be used to assist in therapeutic decision-making (24).

We considered early on that risk conveyed by the mesenchymal subtype may simply be replaced in risk models by a gene expression-based lymph node positivity predictor. To investigate, we constructed a predictor of lymph node positivity similar to those reported by others (4, 25). Briefly, our predictor had an accuracy of 66% in the OC training fit and only 57% in the independent test set, which was comparable to accuracies obtained by Chung et al. (2004) (4) (53%–60%) when OC tumors were included in classifier training. They found that removing OC tumors from training improved classifier performance; however, OC being a focus of our work, we decided to pursue the mesenchymal subtype as a biomarker of outcomes.

In the context of radiation, we considered possible explanations for the poor survival experienced by mesenchymal patients in some strata but not others. The overall inferior survival of patients with EMT signature, the most prominent component of the mesenchymal subtype, has been suggested by many prior reports (8, 21, 22, 26). Mesenchymal tumors are characterized by EMT programs of gene expression, as well as inflammatory signatures that might be associated with worse outcomes. However, such programs might not be expected to have differential outcomes with respect to node-positive versus node-negative disease. One possible explanation would be differential treatment, especially radiation. In node-negative patients, radiation was administered at overall similar rates between mesenchymal and non-mesenchymal patients, 40% and 45% respectively, suggesting that differential radiation alone would not explain differential outcomes. The role of chemotherapy in node-negative patients would be in conjunction with radiation and would only be limited to patients with positive margins, and as such, differential chemotherapy usage is also an unlikely explanation for differential outcomes. As expected, radiation is more common in node-positive patients. Among node-positive patients, mesenchymal patients were radiated at slightly lower rates overall, 62% versus 76%. Similarly, in the non-OC sites (larynx and oropharynx), node-negative mesenchymal and non-mesenchymal patients were radiated at somewhat higher rates, 56% and 49%, respectively, consistent with higher rates of radiation-based treatment of these disease sites. Node-positive non-OC mesenchymal and non-OC, non-mesenchymal patients were radiated at the highest rates of 73% and 88%, respectively, likely a combination of primary chemoradiation and adjuvant radiation cases. Although speculative, it is at least possible that part of the difference between patient groups might be due to the use of radiation, in which the poor prognosis of early-stage OC mesenchymal patients can be at least somewhat attenuated in higher stage compared to non-mesenchymal patients when they are radiated. This would argue that increased radiation of node-negative OC mesenchymal patients might be beneficial.

We believe that while the conclusions of this study are well supported by the data presented, this study was limited by its retrospective nature and unavailability of patient treatment status from TCGA. Furthermore, the study would benefit from the availability of a larger validation dataset.

Predicting recurrence or relapse events in early-stage, HPV-negative HNSCC remains a significant challenge for clinicians. Despite significant improvements in our understanding of HNSCC molecular biology and prognostication, there is a paucity of biomarkers that have been developed to address this specific issue. Our data suggest that a gene expression classifier applied to early-stage HNSCC could potentially be used to assist in therapeutic decision-making.

## Data availability statement

The original contributions presented in the study are included in the article/**Supplementary Material**. Further inquiries can be directed to the corresponding author.

## Ethics statement

The studies involving human participants were reviewed and approved by Institutional Review Boards of Washington University in St. Louis (IRB#201706088) and the University of Tennessee Health Science Center (IRB# 17-05549-XP). The ethics committee waived the requirement of written informed consent for participation.

## Author contributions

JZ, DH, JU, and GM contributed to the study concept and design. GM performed computational analyses. JZ, DH, JU, and GM contributed to the writing and revision of the manuscript. All authors have read and approved the final version of the manuscript.

## Funding

This work was funded by a grant from the Department of Health and Human Services, National Institutes of Health—National Cancer Institute: Grant Number 1R01CA211939-01A1.

## Conflict of interest

JU and GM are employed by GeneCentric Therapeutics, Inc.

The remaining authors declare that the research was conducted in the absence of any commercial or financial relationships that could be construed as a potential conflict of interest.

## Publisher’s note

All claims expressed in this article are solely those of the authors and do not necessarily represent those of their affiliated organizations, or those of the publisher, the editors and the reviewers. Any product that may be evaluated in this article, or claim that may be made by its manufacturer, is not guaranteed or endorsed by the publisher.
