# Supplementary material for: Mesenchymal gene expression subtyping analysis for early-stage human papillomavirus-negative head and neck squamous cell carcinoma reveals prognostic and predictive applications
Source: Front Oncol. 2022 Sep 6;12:954037. doi: 10.3389/fonc.2022.954037 (PMC9486405; doi:10.3389/fonc.2022.954037)
Supplement: Supplementary file 1 [file DataSheet_1.pdf]

Supplementary data

Supplementary Table 1.
88 Gene Set Reduced Centroid Predictor

FAM3B	CDSN	RAB6B	OLFML3
TMPRSS2	GSDMA	ABCC1	PCOLCE
FOXA1	PLD2	TXNRD1	LEPRE1
TJP3	PPARD	CYP26A1	NNMT
MEIS1	FBLIM1	EPCAM	OLFML2B
HLF	DHRS1	CHST7	COL6A2
PLAC8	KIAA1609	ABCC5	PHLDB1
FUT6	ATP6V1D	CABYR	COL6A1
TMPRSS11B	EPGN	RIMKLA	CMTM3
MUC4	MAP7D1	PRKX	GPX8
MUC20	KRT6B	PIR	PTH1R
ACTN1	ZDHHC2	TMEM51	CYP2C18
TGFBI	SGEF	IL4R	GRHL3
APBB2	P4HTM	FAM40A	CSTA
FSTL3	PATZ1	APOL3	ELF3
INHBA	NSUN7	MOBKL2B	SPRR3
SERPINH1	WNK2	SLC31A2	ADH7
SNAI2	GCNT2	CIITA	ALDH3A1
SERPINE1	LTBP3	UBA7	TMPRSS11A
SFXN3	CHPT1	AQP3	KLF5
CAV1	PBX1	CASP4	SLC9A3R1
F2RL1	FAM171A1	SLAMF7	SOX2

88 Gene Reduced Centroid Predictor
